# Science against Common Sense

**Published:** 1874-05

**Authors:** 


### SCIENCE AGAINST COMMON SENSE.

Some years ago a man wrote a book to prove that the cereals, such as wheat and corn and oats and rye, had so much lime in them that if people did not quit using flour, the bones would become so limey, so brittle, that if a man fell down he would break into as many pieces as a cast of plaster of Paris. Later, Mr. Graham came along, and actually persuaded half the people of New England that wheat bread should not be eaten; that it was deprived of all its lime, which was contained in the bran, and that the result in another generation would be that there would be no teeth to eat with; so everybody began to eat bread as black as the pot, instead of the delightful snowy loaves which had adorned our tables for a generation. Then came a cavalcade of jaundiced vegetarians, declaring it a sin and a shame, a perfect barbarism, to be killing all the cattle, curly-tailed pigs, and all the innocent little lambs, and the patient, useful ox, and eating them up like a parcel of cannibals.

Last Summer, while in England, John Bull was kicking up a tremendous row about the danger of drinking milk, that it was all full of bugs and poisons, sure to give everybody typhoid fever. The other day, a scientific gentleman demonstrated in a lecture that the purest water (apparently) that ever bubbled from a spring at the foot of a hill within an hour of the City Hall, had killed off a whole family; for that, sparkling, and pure and limpid as it seemed to be, it gave, on being allowed to rest, a perceptible sediment at the bottom, which, being put under a microscope, showed that it was nothing more than the solid matter from sinks, privies, and house-drains of the families higher up the hill; and that this was the element which had destroyed the family who drank this crystal-like water with typhoid fever. Now, here comes Mr. Mynheer Von all the way from Dutchdom, declaring that the milk of a tubercular cow gives intestinal catarrh, followed by the tubercularization of the mesenteric ganglion, liver and spleen, ending in extensive miliary tuberculosis of the thorascic viscera; all which means that if the old cow is sick, every man and Jack of you will have confirmed consumption in little or no time, if you drink her milk. The water-cure people hold up their hands in holy horror at the very mention of eating pork, pointing triumphantly at millions of trichinæ squirming around in a man's muscles, inflicting pains, second only to those resulting from the rack or the "wheel."

According to all this, we must not eat bread nor meat; we must not drink water nor milk; and whole battalions of people declare that wine and beer and bitters and cordials are an abomination. Others forbid apple pies, plum puddings and sugar candy.

Besides these, omitting crimes, we must commit the still greater ones, of plunging into ice-water every morning, then scrub all the skin off with a horse-hair brush or a coarse board towel; sit down to breakfast of oatmeal sawdust; dine off a tablespoonful of wheat and two berries, and make a supper on catnip tea, then be put through a Russian bath of five hundred degrees; sleep under an open window when the thermometer is at zero, wearing long hair; dress the women in pantaloons; make all our property over to them; then sit down in the kichen corner and nurse the baby, and, when it is asleep, help wash up the tea-things, and go to bed at nine o'clock to be "out of the way." What will become of us men? Surely we have fallen on evil times. A better and truer mode of life is to have plenty of everything that is good to eat and drink, which imparts nourishment and strength, and as much of it as you want. The idea of getting up from the table hungry is unnatural, and absurd, and hurtful—quite as much so as getting up in the morning before your sleep is out, on the mischievous principle that, "early to rise, makes a man healthy, wealthy and wise." Early rising, in civilized society, always tends to shorten life. Early rising of itself never did anybody any good. Many a farmer's boy has been made an invalid for life by being made to get up at daylight, before his sleep was out. Many a young girl has been stunted in body and mind and constitution by being made to get up before the system has had its full rest. All who are growing, all who work hard, and all weakly persons should not get up until they feel as if they would be more comfortable to get up than to remain in bed; that is the only true measure of sufficiency of rest and sleep. Any one who gets up in the morning feeling as if he "would give anything in the world" to remain in bed a while longer, does violence to his own nature, and will always suffer from it—not immediately, it may be, but certainly in later years, by the cumulative ill effects of the most unwise practice.

In any given case, the person who gets up in the morning before he is fully rested, will lack just that much of the energy requisite for the day's pursuit.

As a people, we do not get enough sleep; we do not get enough rest; we will not take time for these things; hence our nervousness, our instability, our hasty temper, and the premature giving out of the stamina of life. Half of us are old at three-score, the very time a man ought to be in his mental, moral and physical prime. Half of our wives, especially in the farming districts, die long before their time, because they do not get rest and sleep proportioned to their labor. Nine times out of ten it would be better for all parties, if the farmer should get up and light the fires and prepare breakfast for his wife, she coming directly from her toilet to the breakfast table; because it almost always happens that she has to remain up to set things right long after the husband has gone to bed, when really he has nothing to do after supper but to go to bed. This is a monstrously cruel imposition on wives and mothers.

